# Prevalence of hypertension and prehypertension in Nepal: a systematic review and meta-analysis

**DOI:** 10.1186/s41256-019-0102-6

**Published:** 2019-04-30

**Authors:** Yun Huang, Pi Guo, Biraj M. Karmacharya, Sharvesh Raj Seeruttun, Dong Roman Xu, Yuantao Hao

**Affiliations:** 1 Department of Medical Statistics and Epidemiology, School of Public Health, Sun Yat-sen University, No. 74 Zhongshan Road II, Guangzhou, 510080 Guangdong Province People’s Republic of China; 2 Department of Preventive Medicine, Shantou University Medical College, Shantou, 515041 Guangdong Province China; 3 Sun Yat-sen Global Health Institute (SGHI), School of Public Health and Institute of National Governance of Sun Yat-sen University, Guangzhou, 510275 Guangdong Province China; 4 Department of Gastric Surgery, Sun Yat-sen University Cancer Center, State Key Laboratory of Oncology in South China, Collaborative Innovation Center for Cancer Medicine, Guangzhou, 510060 Guangdong Province China

**Keywords:** Hypertension, Prehypertension, Prevalence, Nepal, Systematic review, Meta-analysis

## Abstract

**Background:**

Studies regarding blood pressure of Nepal have demonstrated a contrasting prevalence. We aimed at providing a generalized estimate of the prevalence of hypertension and prehypertension in urban, suburban, and rural areas of Nepal.

**Methods:**

This systematic review followed PRISMA guidelines. A thorough search of PubMed, EMBASE, and Web of Science was performed, and studies satisfying the eligibility criteria were reviewed. Pooled prevalence was calculated by random-effects model, and the sources of heterogeneity were explored with meta-regression and subgroup analysis.

**Results:**

Twenty-three studies with 99,792 subjects were identified, and the estimated rate of hypertension and prehypertension were found to be 27.3% (95% CI: 23.8–30.9) and 35.4% (30.3–40.8). The prevalence of hypertension was 28.4% (22.4–34.7), 25.5% (21.4–29.8), and 24.4% (17.9–31.6) among urban, suburban, and rural populations, respectively. Moreover, rates of hypertension were found to be substantially higher in male (31.6%, 27.3–36.1) compared to female (20.0%, 14.2–26.6), and significantly higher among the middle-aged (≥40 years; 36.8%, 29.4–44.5) than among younger adults (< 40 years; 13.2%, 9.2–17.7). Further, prehypertension prevalence was found to be highest in rural areas (40.4%, 25.4–56.4) followed by urban areas (29.3%, 20.8–38.5) and lowest in suburban areas (25.5%, 18.9–32.7).

**Conclusions:**

Our study identified an alarming situation of hypertension among Nepalese males and middle-aged, and a situation of concern with prehypertension in rural areas affecting almost 40 % of the population.

**Electronic supplementary material:**

The online version of this article (10.1186/s41256-019-0102-6) contains supplementary material, which is available to authorized users.

## Background

High blood pressure (HBP) is responsible for more than half of all strokes and coronary disease [1], and is now considered the biggest contributor to the global burden of non-communicable diseases (NCDs) and mortality [2]. The World Health Organization (WHO) reported HBP to be more alarming in low- and middle-income countries (LMICs) [3]; with Nepal being no exception as it is currently suffering from a double burden of diseases due to its transition from a phase of communicable diseases to that of a higher prevalence of NCDs [4].

One study analyzing HBP in developing countries pointed out that the prevalence of hypertension in Nepal was still at a low level (< 20%) [5], in contrast to another study conducted in the Birendranagar municipality of the Surkhet District which indicated a more severe status with an HBP percentage rising as high as 38.9% [6]. This discrepancy may have emerged due to the diverse prevalence of hypertension in different populations of Nepal. Further, the rate of prehypertension, which was also reported notably different from each other (range: 22.1–48.0%) [7, 8], will undeniably evolve to HBP if not controlled . A more generalized estimate of hypertension and prehypertension prevalence will therefore provide an important background to health-related authorities to understand the disease’s status in Nepal. Although there were three nationwide studies aiming at probing the HBP situation in Nepal [9–11], they reported diverging hypertension and prehypertension status which probably arose due to the different age compositions of respondents and other confounders. The actual blood pressure (BP) status in Nepal urges further verification. At present, there has been no systematic review of the Nepalese prehypertension situation. Limited existing meta-analyses for hypertension have been broadly focused either on LMICs, Asian populations, or the South Asian Association for Regional Cooperation (SAARC), but none has been focused specifically on Nepal. In addition, previous studies concentrated on comparing BP status between urban and rural areas [9, 10], while many epidemiological studies were conducted in suburban areas and the prevalence of HBP in this region need to be clarified, and targeted policies may benefit from regional analysis.

Thence, we aimed at filling this gap using strong meta-analytic evidence by merging existing scientific literature to obtain a robust generalized estimate of the prevalence of both hypertension and prehypertension in the urban, suburban, and rural parts of Nepal.

## Methods

### Study design

This systematic review was conducted following PRISMA (Preferred Reporting Items for Systematic Reviews and Meta-Analyses) guidelines [12], and the PRISMA checklist is provided as an Additional file 1. Although the criteria for HBP is evolving, in our study the presence of prehypertension was still defined as systolic BP 120–139 mmHg and diastolic BP 80–89 mmHg; and hypertension was defined as an average BP ≥140/90 mmHg and/or the use of antihypertensive medication according to the JNC VII report [13], because the latest criteria is not widely accepted yet and all the compiled studies complied with the JNC VII report. The method used in this study was composed of the following steps: (1) a survey of the literature for relevant studies on the prevalence of hypertension in Nepal; (2) data extraction; and after pooling data, (3) meta-analysis.

### Search strategy and selection criteria

An initial search for studies by a combination of Medical Subject Headings (MeSH) terms consisting of ‘hypertension’ and its relevant synonyms was performed using PubMed, EMBASE, and Web of Science. The search was restricted to publications from January 2000 to August 2018 that were conducted on the human species and published in the English language. (We also searched relevant databases for possible publications in Nepalese and Chinese but none was found). The results were further narrowed by adding ‘Nepal’ as another key word. The search details of PubMed were as follows: ((“hypertension”[MeSH Terms] OR “hypertension”[All Fields]) OR “high blood pressure”[All Fields] OR “elevated blood pressure”[All Fields] OR “raised blood pressure”[All Fields]) AND (“nepal”[MeSH Terms] OR “nepal”[All Fields]) AND ((“2000/01/01”[PDAT]: “2018/08/31”[PDAT]) AND “humans”[MeSH Terms] AND English[lang]). A manual search for additional potential studies was performed using the references cited in the retrieved reviews and original research articles. The reason why excluding studies conducted before year 2000 was the definition of ‘hypertension’ we use being lastly revised and implemented in year 1999.

**Fig. 1:**
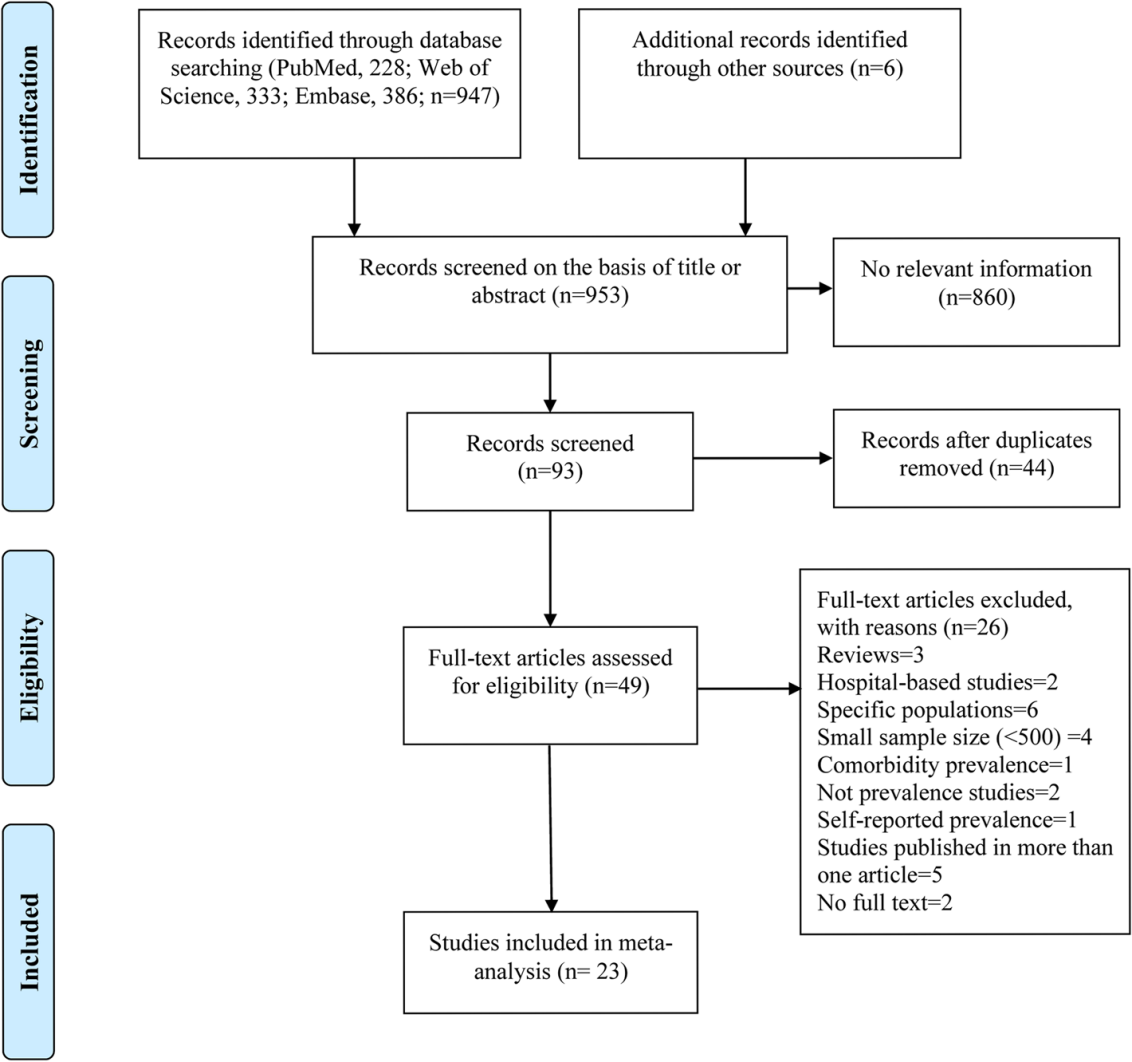
PRISMA flow diagram of the study selection process

Two authors (Yun Huang, Pi Guo) independently reviewed the titles and abstracts culled from the searches, and full texts of potentially eligible studies were downloaded and further screened for final inclusion in our study. The eligibility criteria for inclusion were: (1) original articles from non-hospitalized and population-based surveys reporting HBP prevalence (or containing data to calculate the prevalence); (2) respondents without restriction to specific age groups or populations; (3) containing information on the study location (urban/suburban/rural); (4) presenting HBP prevalence without other associated comorbidities; (5) using a non-convenience sampling method and with a sample size above 500 participants. For studies published in more than one edition, we considered the most comprehensive one. When there was uncertainty or disagreement between the two authors as to the eligibility of a study, another author (Yuantao Hao) was asked for guidance to reach a consensus. The study selection process is shown in Fig. 1.

### Data extraction

All data were extracted independently by two authors (Yun Huang, Pi Guo) using a standardized protocol. The characteristics recorded for each eligible study included the first author’s name, year of publication, study design, sampling methods, age range of participants, net sample size (the total number of participants, and the number of male and female separately), number of events (hypertensive and pre-hypertensive cases), corresponding prevalence, methods of BP measurement (the type of device used, the number of BP readings taken, and the time interval between the measurements), diagnostic criteria of hypertension, response rate, survey year, survey site, and location of study (urban/suburban/rural).

We then performed a quality assessment of the retained studies based on the completeness of the eligibility criteria, sampling strategy, age range, sample size, non-response rate, survey year, explanation of limitations of the study, and BP measurement techniques [14, 15]. In total, twelve domains were assessed. A score of 1 was allocated for those fulfilling the conditions in each domain, a score of 0.5 for partial fulfilment, and a score of 0 for non-fulfillment of the conditions. The detailed scoring table is provided as a Additional file 2. The maximum score was 12 and studies scoring 10.0 or above were considered high-qualified, those scoring 8.0–9.5 were classified as middle-qualified, and the rest were classed as low-qualified.

### Statistical analysis

Meta-analysis was performed using the packages ‘meta’ and ‘metafor’ in R software (version 3.4.2). To weaken the effect of studies with extremely small or large prevalence on the overall estimate, we transformed the data with the Freeman-Tukey double arcsine function before pooling the prevalence [16]. We estimated heterogeneity among studies using Cochrane’s chi-square (*χ2*) test and quantified it with *I*^2^ index. *I*^2^ is defined as the proportion of total variation provided by between-study variation, and was denoted the values of 0, 25, 50, and 75% which corresponds to no, low, moderate, and high heterogeneity, respectively [17]. A *p* < 0.05 from Cochrane’s chi-square (*χ2*) test or large *I*^2^ demonstrate substantial heterogeneity. Random-effects model was constructed to address heterogeneity in the pooled prevalence. An univariate meta-regression was performed by setting possible confounders as moderator with function ‘rma.glmm’ in the ‘metafor’ package to explore for sources of heterogeneity (a 5% level of statistical significance), then we categorized studies into subgroups according to findings from the meta-regression and performed meta-analysis for each subgroup respectively to certify the results of the meta-regression. We applied the symmetry of funnel plots and did both Begg’s adjusted-rank correlation test and Egger’s regression asymmetry test to evaluate publication bias [18, 19]; a *p*–value of less than 0.10 was considered indicative of statistically significant publication bias.

## Results

### Study characteristics

In total, 953 studies were retrieved, of which 23 met the inclusion criteria in the primary review of hypertension prevalence [6–11, 20–36]. It should be noted that one researcher (Sharma S.K.) had two publications that analyzed overlapping populations [23, 25]. In the 2010 publication we only extracted pre-hypertensive data as this was not presented in the second report, and only hypertensive data were extracted from the 2011 publication. Three other studies conducted among specific gender groups were only included in the gender-specific subgroup analysis [21, 27, 30].

As can be observed from Table 1, the publication years spanned from 2006 to 2018. The 23 retained studies totaled 99,792 participants (ranging from 527 to 15,934 participants). In the retained studies, the majority of participants were found to be above 18 years of age. The response rates of each survey varied from 69.2 to 99.6%. Excepting two studies in which the surveyed time was not reported, eleven studies were conducted after 2010 and the other ten studies were conducted between 2001 to 2010. The surveyed sites covered Nepal broadly, among which six were from urban regions, six from suburban regions, six from rural areas, and five contained both urban and rural residents. In most of the studies analyzed, a manual sphygmomanometer (*n* = 12) was preferred, nine others used a digital sphygmomanometer, and two did not provide any information about the measuring device used. The frequency for taking BP measurements varied from one to four and the intervals between each measurement ranged from half a minute to half an hour. Each study was scored and ranked accordingly; out of these only three studies were identified as low-qualified [8, 30, 32].

**Table 1:** Study characteristics

Study ID	Study design	Sampling method	Age range	Sample size(Male/Female)	Number of events		Prevalence		BP measurement	Frequency of measurement	Interval for BP measurement(min)	Hypertension definition	Response rate	Survey year	Survey site	Area	Quality
					Hypertension(Male/Female)	Prehypertension(Male/Female)	Hypertension(Male/Female)	Prehypertension(Male/Female)									
Deewakar Sharma (2006)	Cross-sectional study	Systematic random sampling	18–97	1114 (541/573)	219 (120/99)	246	19.7 (22.2/17.3)	22.1 (NR)	Manual	2	NR	≥ 140/90 OR medicine	90.64	Feb to Jun 2005	Southern Kathmandu Valley	suburban	high
U. K. Shrestha (2006)	Cross-sectional study	Cluster sampling	≥ 40	1012 (423/589)	236 (99/137)	NR	23.3 (23.4/23.3)	NR	Manual	2	0.5	≥ 140/90	85.7	2001 to 2002	Seven urban municipalities	urban	high
Vaidya A (2007)^a^	Cross-sectional study	Simple random sampling	≥ 35	1000 (1000/0)	227 (227/−)	NR	- (22.7/−)	NR	Manual	2	5	≥ 140/90 OR medicine	NR	2004 to 2005	Dharan Municipality	urban	middle
Koju R (2010)	Cross-sectional study	Systematic random sampling	18–88	796 (306/490)	230 (88/142)	232	28.9 (28.8/29)	29.1 (NR)	Manual	2	30	≥ 140/90 OR medicine	69.22	2007	Dhulikhel Municipality	suburban	high
Sanjib Kumar Sharma (2010)^b^	Cross-sectional study	NR	18–97	8397 (3199/5185)	3009 (1341/1668)	3230 (1247/1983)	36 (42/32)	39 (39/38)	Manual	1	NR	≥ 140/90 OR medicine	NR	2007	Dharan\Tarahara\Damak\Biratnagar\Birtamod	urban	middle
Sanjib Kumar Sharma (2011)	Cross-sectional study	NR	20–100	14,422 (5327/8679)	4894 (2164/2603)	NR	34 (40.7/30)	NR	Manual	1	NR	≥ 140/90 OR medicine	NR	2007	Dharan\Tarahara\Damak\Biratnagar\Birtamod	urban	middle
KD Mehta (2011)	Cross-sectional study	NR	≥ 30	1938 (NR)	615 (NR)	752	31.7 (NR)	38.8 (NR)	Manual	2	5	≥ 140/90	93.85	Sep 2005 to Jul 2006	Sunsari District	both	middle
Chataut J (2011)	Cross-sectional study	NR	≥ 18	527 (214/313)	118 (70/48)	253	22.4 (32.7/15.3)	48 (NR)	Manual	2	NR	≥ 140/90	NR	NR	Bolde (a rural village in hilly region of central part of Nepal)	rural	low
Abhinav Vaidya (2012)	Cross-sectional study	NR	≥ 21	1218 (527/691)	412 (202/213)	NR	33.8 (38.3/30.8)	NR	Manual	1	NR	≥ 140/90	84	2006	Bhadrabas village area of Kathmandu Valley	rural	high
Rumana J Khan (2013)^a^	Cross-sectional study	NR	16.4–71.2	15,934 (0/15934)	530 (−/530)	2296	- (−/3.3)	-(−/14.4)	Digital	4	NR	≥ 140/90	96.75	2006 to 2008	Sarlahi District	rural	middle
Abhinav Vaidya (2013)	Cross-sectional study	Kish technique	25–59	777 (229/548)	168 (NR)	NR	21.6 (NR)	NR	Digital	3	5	≥ 140/90 OR medicine	93.92	Sep to Nov 2011	JD-HDSS of Bhaktapur District in the Kathmandu Valley	suburban	high
Sanjib Kumar Sharma (2013)	Cross-sectional study	NR	≥ 20	3218 (1542/1676)	1243 (NR)	NR	38.6 (NR)	NR	NR	NR	NR	≥ 140/90 OR medicine	NR	2003 to 2005	Four VDCs in Dharan Municipality	rural	middle
Adhikari K (2014)	Cross-sectional study	Systemic random sampling	15–64	1240 (665/575)	276 (165/111)	510 (285/225)	22.3 (24.8/19.3)	41.1 (42.8/39.1)	NR	NR	NR	≥ 140/91 OR medicine	NR	Mar to Jun 2013	Six urban and six rural districts	both	middle
Bhandari S (2014)^a^	Interventional study	NR	15–49	14,300 (0/14300)	948 (−/948)	NR	- (−/6.6)	NR	Manual	NR	NR	≥ 140/90 OR medicine	NR	2011 to 2012	VDCs of nine districts	rural	low
Koju R (2015)	Cross-sectional study	Multistage random cluster sampling	18–65	2100 (861/1239)	317 (187/130)	915	15.1 (21.7/10.5)	43.6 (NR)	Digital	2	2	≥ 140/90	99.6	May-13	Nationwide	both	high
Chataut J (2015)	Cross-sectional study	NR	≥ 18	648 (258/390)	133 (79/54)	302 (115/187)	20.5 (30.6/13.8)	46.6 (44.5/47.9)	Manual	NR	NR	≥ 140/90 OR medicine	NR	NR	Ramechap District	rural	low
Krishna Kumar Aryal (2015)	Cross-sectional study	Multistage random cluster sampling	15–69	4143 (1336/2807)	1065 (415/578)	NR	25.7 (31.1/20.6)	NR	Digital	3	3	≥ 140/90 OR medicine	98.2	Jan to June 2013	Nationwide	both	high
Raja Ram Dhungana (2016)	Cross-sectional study	Systematic random sampling	18–70	587 (242/345)	191 (93/98)	NR	32.5 (38.4/28.4)	NR	Manual	3	3	≥ 140/90 OR medicine	97.8	Jan to Jul 2015	Kageshwari-Manohara and Nagarjun Municipalities	urban	middle
Biraj M Karmacharya (2017)	Cross-sectional study	Simple random sampling	≥ 18	1073 (446/627)	298 (167/131)	NR	27.8 (37.4/20.9)	NR	Digital	3	NR	≥ 140/90 OR medicine	NR	Nov 2013 to Feb 2015	Dhulikhel Municipality	suburban	high
Dinesh Neupane (2017)	Cross-sectional study	Kish technique	25–65	2815 (971/1844)	838 (369/424)	NR	29.8 (38/23)	NR	Digital	3	NR	≥ 140/90 OR medicine	98	2013	Lekhnath Municipality	suburban	high
Mahesh Kumar Khana (2017)	Cross-sectional study	Multistage clustered sampling	≥ 30	1159 (335/824)	451 (161/290)	NR	38.9 (48.1/35.2)	NR	Digital	2	3	≥ 140/90 OR medicine	96.6	Jan to Dec 2016	Birendranagar Municipality of Surkhet District	Urban	High
Ministry of Health,Nepal (2017)	Cross-sectional study	Two stage stratified cluster sampling	≥ 15	14,494 (6059/8435)	2835 (1418/1417)	3961 (1903/2058)	19.6 (23.4/16.8)	27.3 (31.4/24.4)	Digital	3	5	≥ 140/90 OR medicine	99	Jun 2016 to Jan 2017	Nationwide	both	high
Basant Maharjan (2018)	Cross-sectional study	Proportionate probability sampling	20–59	580 (264/316)	215 (110/105)	130	37.0 (41.6/32.2)	22.4 (NR)	Digital	NR	NR	≥ 140/90 OR medicine	NR	Dec 2015 to Apr 2016	Kirtipur Municipality	urban	middle

^a^The study conducted among specific gender groups was only included in the gender-specific subgroup analysis.^b^The study contained overlapped respondents with another study (Sanjib Kumar Sharma 2011), and was only included in the pre-hypertensive prevalence estimation

*NR* not reported

—: not applicable

### Burden of hypertension and prehypertension

**Fig. 2:**
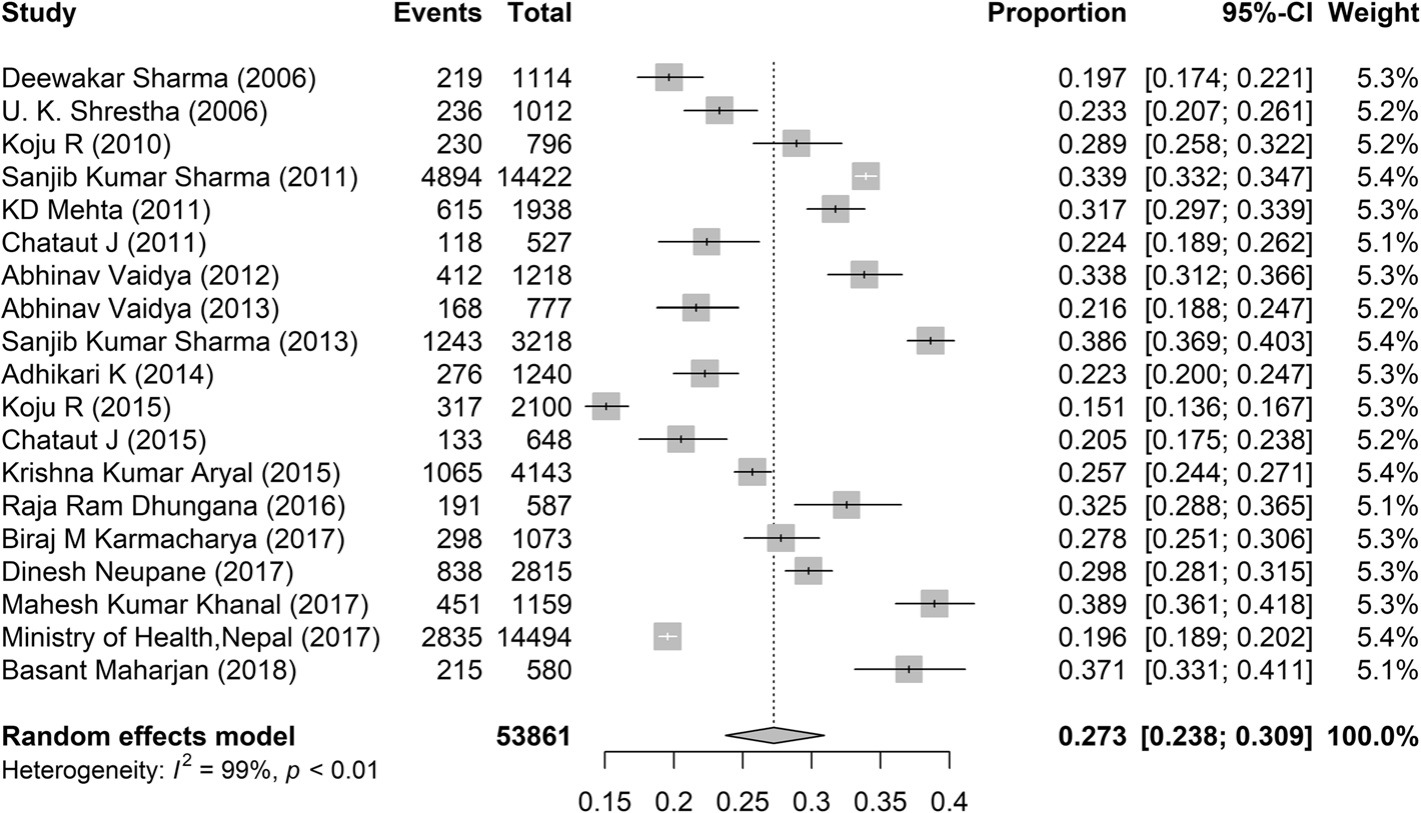
Prevalence of hypertension in Nepal

The HBP situation in Nepal is illustrated by the Forest plot in Fig. 2 Previous reports of HBP prevalence were found to vary widely, with rates ranging from 15.1 to 38.9%. It is worth mentioning that seven out of nineteen studies showed prevalence higher than 30% and that the overall estimate was found to be 27.3% (95% confidence interval (CI): 23.8–30.9%).

The pooled regional estimates of hypertension were 28.4% (95% CI: 22.4–34.7%), 25.5% (95% CI: 21.4–29.8%), and 24.4% (95% CI: 17.9–31.6%) for the urban, suburban, and rural areas respectively. HBP was found to be substantially affecting male (31.6%; 95% CI: 27.3–36.1%) as compared to female (20.0%; 95% CI: 14.2–26.6%), and this tendency was found to be consistent in three residential areas (Table 2).

**Table 2:** Prevalence of hypertension in Nepal classified by area, age, and gender

Characteristics	Male	Female	Both genders
Residence			
Urban	33.8 (26.3, 41.8)	27.6 (20.9, 34.8)	28.4 (22.4, 34.7)
Suburban	31.5 (23.8, 39.7)	22.4 (18.5, 26.5)	25.5 (21.4, 29.8)
Rural	30.2 (20.5, 40.9)	13.1 (7.7, 19.6)	24.4 (17.9, 31.6)
Age (years)			
< 30	7.0 (6.0,8.2)	2.8 (2.1,3.4)	8.1 (4.9,12.0)
30–39	24.4 (15.3,34.8)	13.4 (9.5,17.9)	16.2 (13.8,18.7)
40–49	32.6 (19.3,47.4)	29.1 (19.5,39.8)	28.9 (22.4,35.8)
50–59	44.9 (27.7,62.7)	39.4 (26.6,53.0)	35.6 (26.8,45.0)
≥ 60	44.7 (33.7,55.9)	44.7 (33.3,56.5)	43.5 (33.8,53.5)
< 40	19.9 (9.0,33.7)	10.7 (4.6,19.0)	13.2 (9.2,17.7)
≥ 40	41.8 (29.6,54.4)	37.9 (28.0,48.4)	36.8 (29.4,44.5)
Total	31.6 (27.3, 36.1)	20.0 (14.2, 26.6)	27.3 (23.8, 30.9)

**Fig. 3:**
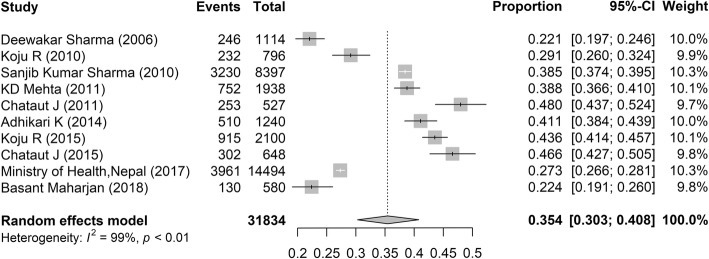
Prevalence of prehypertension in Nepal

Ten studies reported the prevalence of prehypertension [7, 8, 10, 11, 22–24, 31, 32, 36], among which, the lowest and highest rate was 22.1 and 48.0%, and the pooled estimate was 35.4% (Fig. 3; 95% CI: 30.3–40.8%). In addition, the prevalence of prehypertension was calculated to be highest in rural areas (40.4, 95% CI: 25.4–56.4%), followed by urban areas (29.3, 95% CI: 20.8–38.5%), and lowest in the suburban areas (25.5, 95% CI: 18.9–32.7%). And there was no significant difference between male (39.0, 95% CI: 33.1–45.2%) and female (37.0, 95% CI: 27.1–47.6%).

### Publication bias and sources of heterogeneity

**Fig. 4:**
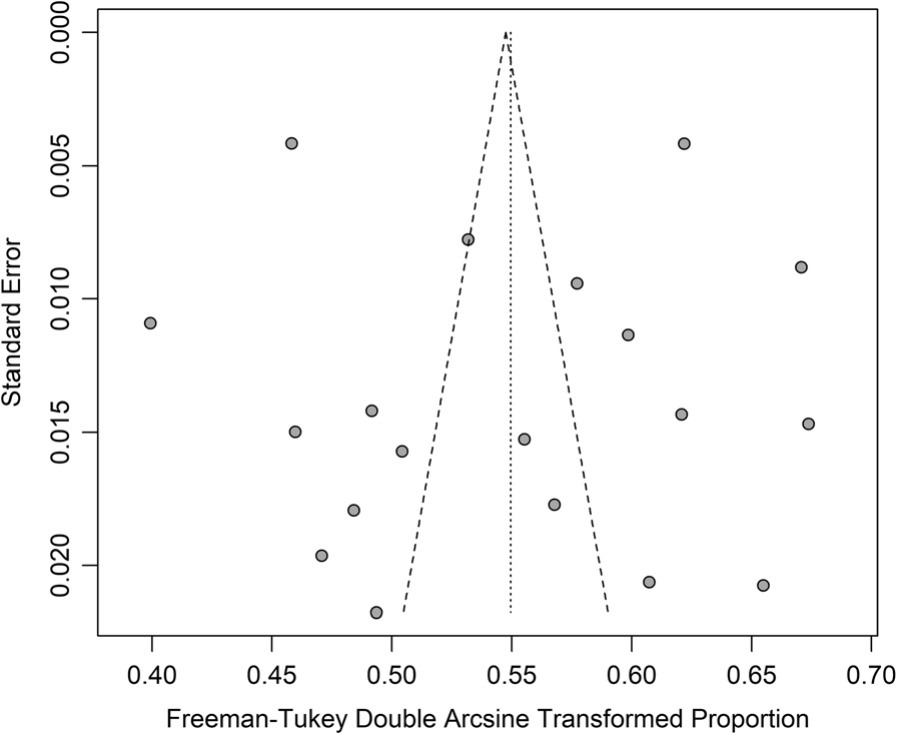
Funnel plot showing the transformed proportion of hypertension in each study by the standard error

Figure 4 illustrates the Funnel plot for the visual assessment of publication bias and, as observed by the symmetrical pattern, no publication bias was found. In addition, both Begg’s adjusted-rank correlation test and Egger’s regression asymmetry test showed no evidence of substantial publication bias (*P* = 0.861 for Begg’s test; *P* = 0.875 for Egger’s test).

Previous results showed a considerable heterogeneity (*I*^*2*^ = 99% and *P* < 0.01) among retained studies. Meta-regression findings indicated that sample size (*P* = 0.928), gender ratio (*P* = 0.948), inception of survey (*P* = 0.721), definition of hypertension (*P* = 0.363), quality score of study (*P* = 0.347), and measurement device (*P* = 0.769) were not associated with the heterogeneity observed (Table 3). Moreover, the age of participants (*P* = 0.001) and quality grade of each study we ranked (*P* = 0.027) were identified as potential sources of heterogeneity.

**Table 3:** Results of the meta-regression model

Moderators	Coefficient	Standard error	*P*-value
Mean age	0.069	0.022	0.001*
Sample size	0.000	0.000	0.928
Gender (male ratio)	−0.078	1.191	0.948
Inception of survey	−0.007	0.018	0.721
Definition of hypertension			0.363
≥ 140/90 OR medicine	Reference		
≥ 140/90	−0.167	0.183	0.363
Quality score of study	−0.057	0.061	0.347
Quality grade			0.027*
middle	Reference		
high	−0.351	0.155	0.023*
low	−0.568	0.255	0.026*
Measurement device			0.769
manual	Reference		
digital	−0.0497	0.1696	0.769

*P < 0.05

To certify the findings of meta-regression, subgroup analyses for specific age and quality grade groups were performed. The HBP prevalence was found to rise with an increase in age and was significantly higher among age group 40 or above (36.8, 95% CI: 29.4–44.5%) in contrast to that below 40 years (13.2, 95% CI: 9.2–17.7%; Table 2). Considering data limits, we just performed subgroup analyses for high- (22.6, 95% CI: 16.4–29.5%), middle- (38.6, 95% CI: 36.9–40.3%), and low- (21.4, 95% CI: 19.1–23.7%) qualified studies within rural areas. It is noticeable that HBP prevalence among middle-qualified studies was actually higher than rates among high-qualified and low-qualified studies.

## Discussion

The pooled prevalence of HBP obtained from our study was found to be 27.3% (95% CI: 23.8–30.9%), higher than that reported in low-income countries (23.1%), but similar to the average rate in the SAARC regions (27.1%) and that reported in the Nepal nationwide STEPS survey (25.7%) [9, 37, 38]. A previous systematic review performed by Neupane D, et al. demonstrated partly comparable results with ours (29.7%; 95% CI: 26.9–32.6%) [38]. However, there are some notable differences. First, Neupane D, et al. aimed at the entire SAARC region’s HBP prevalence and its associated risk factors rather than primarily focusing on the HBP prevalence of Nepalese as presented in our study. Second, they only included five original researches into their meta-analysis, which totaled nearly one-quarter of our participants (*n* = 22,939) and one of their included studies accounted for males only was not representative of Nepalese in general. Thus, a more comprehensive analysis was necessary to reveal the present Nepalese HBP situation.

Previous literature on HBP prevalence often categorized residents by urban and rural regions, while we subdivided residents into three categories, namely urban, suburban, and rural, for an improved practical and factual analysis. Based on the regional analysis, although the HBP status appeared somewhat more severe in urban regions, we found that the prevalence of the three areas was comparable, and this was in accordance with an insignificant difference between urban and rural habitation presented in previous studies [9, 10]. The primary driver for this observed phenomenon may be rapid urbanization, change in dietary patterns, and behavioral factors like smoking and harmful drinking that result in suburban and rural residents catching up with urban populations in the process of exacerbating NCDs [39].

Higher HBP prevalence in male compared to female was found in our study, which was similar to that of two previous systematic reviews [37, 38]. It is worth mentioning that there were only two studies [20, 22], out of the seventeen which mentioned prevalence in both male and female, that reported no considerable differences between the genders. In addition, our findings also revealed that higher HBP in male was consistent in urban, suburban, and rural areas. This gender difference may be attributed to male as they are more easily exposed to behavioral risk factors, such as a significantly high level of tobacco and alcohol use [31].

The pooled prehypertension prevalence in our findings was similar to the estimated global figure (35.4% vs. 38.0% respectively) while higher than that of the SAARC region (29.6%) [38, 40]. Furthermore, results of this meta-analysis identified that the prevalence of prehypertension is more alarming in Nepalese rural populations (40.4%), with the status in urban residents (29.3%) concerning as well, while suburban residents comparably retain a more “comfortable” status (25.5%). We did not find significant differences between male and female as previous reviews did [38, 40]. It is undeniable that prehypertension would probably lead to a considerable burden of hypertension in the coming future. Thus, an urgent need for prioritizing its status is strongly recommended. The findings of this study provide crucial information for Nepalese local authorities to distinguish where to focus awareness and screening programs.

The reported HBP prevalence varied considerably across the included studies indicated a notable heterogeneity. The results of meta-regression and subgroup analyses suggested that heterogeneity was directly associated with the age of participants and the quality grade of the included studies. Further, we found that the prevalence was significantly higher in the middle-aged (≥40 years) compared to that in younger adults (< 40 years). Based on the wide and multi-database literature-search , we are the first to provide strong evidence for such an age boundary. A reasonable explanation was given by a previous study which suggested that age-related structural changes in blood vessels gradually lead to narrowing of the vascular lumen, and consequently could increase the risk of acquiring HBP [41]. For studies conducted in rural areas, middle-quality studies showed a significantly higher prevalence. As to the heterogeneity resulting from respondents’ age, we recalculated the HBP prevalence, with the exclusion of three studies, that included a non-generalized age range without younger adults [6, 20, 24], and the estimate was found to decrease slightly from 27.3 to 26.5% (95% CI: 22.7–30.6%), this may be a more precise figure reflective of present Nepalese hypertension status.

It is worth mentioning that Nepal has developed the national Multi-sectoral Action Plan for prevention and control of NCDs (2014–2020), including hypertension prevention and management for the prevention of cardiovascular disease [42]. One of the programs under the action plan is the WHO Package of Essential Non-communicable disease interventions for primary health care in low-resource settings, which began implementation in Nepal in 2016. The program will cover 75 districts in a 5-year period in Nepal and will continue providing access to the diagnostic services in primary health care settings [43]. These plans may bring feasible ways of mitigating the burden of NCDs.

To our knowledge, this is the first comprehensive report targeted at evaluating the scientific literature on the prevalence of hypertension and prehypertension in the urban, suburban, and rural populations of Nepal. Despite such findings, there are some limitations of this study that should be mentioned. Studies from only three of the most commonly used platforms (PubMed, EMBASE, and Web of Science) were searched. Several included articles provided only a crude prevalence with no specified hypertensive events. Further, a noteworthy heterogeneity was observed among retained studies so that large-scale nationwide and more representative epidemiological studies are needed to confirm the results of this study.

## Conclusion

This systematic review identified an alarming situation of hypertension in Nepalese male and middle-aged (≥40 years), as well as a situation of concern regarding the prehypertension status which affects almost 40 % of the rural population. Improving the population-level awareness of HBP; facilitating routine screening aiming at those high-risk groups through integrating of NCDs with primary health care; and effective implementation of NCDs Multisectoral Action Plan (2014–2020) offer potential means for addressing the burden of increased blood pressure in Nepal.

## Additional files

Additional file 1: Prisma Checklist. (DOC 64 kb)

Additional file 2: Scoring table for quality assessment of the retained studies. (DOCX 14 kb)

## Abbreviations

BP: Blood pressure
CI: Confidence interval
HBP: High blood pressure
LMICs: Low- and middle-income countries
MeSH: Medical Subject Headings
NCDs: Non-communicable diseases
PRISMA: Preferred Reporting Items for Systematic Reviews and Meta-Analyses
SAARC: South Asian Association for Regional Cooperation
WHO: World Health Organization
