# Supplementary material for: Prevalence of hypertension and prehypertension in Nepal: a systematic review and meta-analysis
Source: Glob Health Res Policy. 2019 Apr 30;4:11. doi: 10.1186/s41256-019-0102-6 (PMC6489280; doi:10.1186/s41256-019-0102-6)
Supplement: Supplementary file 2 — Scoring table for quality assessment of the retained studies. (DOCX 14 kb) [file 41256_2019_102_MOESM2_ESM.docx]

**Scoring table for quality assessment of the retained studies**

| **Study characteristics** | **Score** |
| --- | --- |
| 1. Original articles from non-hospitalized and population-based surveys reporting on HBP prevalence (or contained data to calculate the HBP prevalence) | One of the eligibility criteria; all retained studies were scored 1. |
|  |  |
| 2. Respondents without restriction to specific age groups or populations | One of the eligibility criteria; all retained studies were scored 1. |
| 3. Containing information on the study location (urban/suburban/rural) | One of the eligibility criteria; all retained studies were scored 1. |
| 4. Presenting HBP without any associated comorbidities | One of the eligibility criteria; all retained studies were scored 1. |
| 5. Non-convenience sampling method and with a sample size above 500 | One of the eligibility criteria; all retained studies were scored 1. |
| 6. Sampling strategy | 1 for those reporting; 0 for those not reporting it. |
| 7. Age range | 1 for those ranging from youth to old; 0.5 for those not mentioned. |
| 8. Sample size | 1 for sample size above 1000; 0.5 for those between 500 and 1000. |
| 9. Non-response rate | 1 for those reported; 0 for those not reported; 0.5 for those that could be calculated. |
|  |  |
| 10. Survey year | 1 for those reported; 0 for those not reported. |
| 11. Explaining limitations of the study | 1 for those reported; 0 for those not reported |
| 12. Blood pressure measurement techniques | 1 for those detailing the measuring tools, the time intervals, and the frequency of measurement above 2; 0.5 for those detailing the measuring tools and the frequency of measurement above 2; 0 for others. |
|  |  |
